# Cytolethal Distending Toxin Promotes Replicative Stress Leading to Genetic Instability Transmitted to Daughter Cells

**DOI:** 10.3389/fcell.2021.656795

**Published:** 2021-05-07

**Authors:** William Tremblay, Florence Mompart, Elisa Lopez, Muriel Quaranta, Valérie Bergoglio, Saleha Hashim, Delphine Bonnet, Laurent Alric, Emmanuel Mas, Didier Trouche, Julien Vignard, Audrey Ferrand, Gladys Mirey, Anne Fernandez-Vidal

**Affiliations:** ^1^Toxalim (Research Centre in Food Toxicology), Université de Toulouse, INRAE, ENVT, INP-Purpan, UPS, Toulouse, France; ^2^IRSD, Université de Toulouse, INSERM, INRAE, ENVT, UPS, Toulouse, France; ^3^MCD, Centre de Biologie Intégrative, Université de Toulouse, CNRS, UPS, Toulouse, France; ^4^Department of Internale and Digestive Diseases, Pole Digestif, CHU Toulouse, Paul Sabatier University, Toulouse, France; ^5^Unité de Gastroentérologie, Hépatologie, Nutrition, Diabétologie et Maladies Héréditaires du Métabolisme, Hôpital des Enfants, CHU de Toulouse, Toulouse, France

**Keywords:** cytolethal distending toxin, replicative stress, genetic instability, DNA bridge, DNA damage, human colorectal organoid

## Abstract

The cytolethal distending toxin (CDT) is produced by several Gram-negative pathogenic bacteria. In addition to inflammation, experimental evidences are in favor of a protumoral role of CDT-harboring bacteria such as *Escherichia coli*, *Campylobacter jejuni*, or *Helicobacter hepaticus*. CDT may contribute to cell transformation *in vitro* and carcinogenesis in mice models, through the genotoxic action of CdtB catalytic subunit. Here, we investigate the mechanism of action by which CDT leads to genetic instability in human cell lines and colorectal organoids from healthy patients’ biopsies. We demonstrate that CDT holotoxin induces a replicative stress dependent on CdtB. The slowing down of DNA replication occurs mainly in late S phase, resulting in the expression of fragile sites and important chromosomic aberrations. These DNA abnormalities induced after CDT treatment are responsible for anaphase bridge formation in mitosis and interphase DNA bridge between daughter cells in G1 phase. Moreover, CDT-genotoxic potential preferentially affects human cycling cells compared to quiescent cells. Finally, the toxin induces nuclear distension associated to DNA damage in proliferating cells of human colorectal organoids, resulting in decreased growth. Our findings thus identify CDT as a bacterial virulence factor targeting proliferating cells, such as human colorectal progenitors or stem cells, inducing replicative stress and genetic instability transmitted to daughter cells that may therefore contribute to carcinogenesis. As some CDT-carrying bacterial strains were detected in patients with colorectal cancer, targeting these bacteria could be a promising therapeutic strategy.

## Introduction

The cytolethal distending toxin (CDT) was first identified in 1988 in *Escherichia coli* (*E. coli*) strains isolated from patients with diarrhea (Johnson and Lior, 1988a, b). To date, around 30 proteobacteria, including *E. coli*, *Campylobacter jejuni* (*C. jejuni*), *Aggregatibacter actinomycetemcomitans* (*A. actinomycetemcomitans*), *Helicobacter hepaticus* (*H. hepaticus*), or *Haemophilus ducreyi* (*H. ducreyi*), were identified to produce this virulence factor [for review, see Scuron et al. (2016)]. The genital, urinary, and digestive tracts constitute the main niches where CDT-producing bacteria were found. The mechanism of CDT intoxication was characterized by nuclear and cytoplasmic enlargement of mammalian cells giving its name to the toxin (Pérés et al., 1997; Sugai et al., 1998; Blazkova et al., 2010). CDT is a heterotrimeric complex belonging to the AB2-type genotoxin, composed of three subunits, mostly CdtA, CdtB, and CdtC. CdtA and CdtC constitute the regulatory subunits and CdtB the catalytic subunit exhibiting phosphatase and DNase activities, the latter responsible for DNA break formation [for review, see Guerra et al. (2011); Jinadasa et al. (2011)]. It was initially reported that CDT induces direct DNA double-strand break (DSB) in mammalian cells (Frisan et al., 2003). However, further investigations demonstrated that low doses of CDT first induce single-strand breaks (SSB), later converted into DSB during the S phase (Fedor et al., 2013), which may be due to replicative fork inhibition and the induction of a replicative stress. Moreover, at the molecular level, CDT-induced DNA damage activates the ataxia–telangiectasia and Rad-3-related kinase (ATR) and ataxia–telangiectasia mutated kinase (ATM) that initiate the DNA damage repair pathway through the spreading of H2AX phosphorylation (defined as γH2AX) around the DNA lesions (Cortes-Bratti et al., 2001; Fahrer et al., 2014). Then, the following checkpoint kinases CHK1, CHK2, and p53 phosphorylation mediate cell cycle arrest at the G1/S and/or G2/M transitions, depending on cellular host p53 status, allowing DNA-repairing machinery to correct DNA damaging insults (Cortes-Bratti et al., 2001; Alaoui-El-Azher et al., 2010; Fahrer et al., 2014). Homologous recombination (RH), non-homologous end joining (NHEJ), Fanconi anemia (FA), and single-strand break repair (SSBR) pathways were depicted as the main mammalian repair mechanisms involved in the resistance to CDT intoxication to preserve the DNA integrity (Bezine et al., 2016). In case of massive unrepaired or misrepaired DNA damage, senescence or cell death by apoptosis is activated (Cortes-Bratti et al., 2001; Alaoui-El-Azher et al., 2010; Guerra et al., 2011; Jinadasa et al., 2011).

Cytolethal distending toxin has been associated to several diseases. In addition to inflammation, some *in vitro* and *in vivo* experiments support its involvement in cancer. CDT-producing *E. coli* are detected in 15.8% of patients with colorectal cancer while it is not detected in the non-cancer group (Buc et al., 2013). In murine models, CDT produced by *H. hepaticus* or *C. jejuni* enhances inflammation and promotes liver and intestinal tumorigenesis through CdtB (Ge et al., 2007, 2017; He et al., 2019). Moreover, precancerous human colon epithelial cells or rat embryonic fibroblasts chronically exposed to CDT from *E. coli*, *H. ducreyi*, or *H. hepaticus* exhibit cancer hallmarks, such as anchorage-independent growth and genetic instability. Indeed, enhanced frequency of mutagenesis, chromosomal aberrations, interphase and anaphase bridges, and micronuclei are observed in cells chronically intoxicated with CDT genotoxin (Guidi et al., 2013; Graillot et al., 2016).

These studies, relying on chronic infection of mice or cell lines with CDT-producing bacteria or intoxication with purified holotoxins, demonstrate the carcinogenic potential of CDT. However, they did not directly assess the mechanism at the root of genomic instability induced by CDT that supports cancer development, including the impact of CDT on the DNA replication program, the characterization of genetic alterations, and their fate in daughter cells. Ultimately, this approach will allow for a better understanding of CDT cellular target considering its proliferation status. To address these issues, we analyzed the direct consequences of CDT on the DNA replication process after acute exposure to CDT holotoxins in human cells. Both HeLa cells, widely manipulated to study CDT, and the well-characterized U2OS cell line for the analysis of fragile site expression were employed to study the molecular mechanism of CDT intoxication. In addition, RKO colorectal cell line and human colorectal organoids were used to investigate the physiological impact of CDT. Here, we report a slowing down of DNA replication velocity depending on CdtB catalytic activity, mainly in the late S phase. This effect was associated with fragile site expression, accumulation of chromosomal aberrations and chromatin bridges in daughter cells. Finally, we show that CDT holotoxin carries out its genotoxic activity especially in cycling cells of human colorectal organoids leading to defective growth. Collectively, these data suggest that highly proliferating cells could be more sensitive to CDT through induction of a replicative stress favoring the establishment of genomic instability transmitted to daughter cells and associated with tumor progression.

## Materials and Methods

### Cell Lines and Treatments

HeLa, U2OS, and RKO human cells were cultured in Dulbecco’s modified Eagle’s medium (DMEM, Gibco, Life Technologies) supplemented with 10% heat-inactivated calf serum and 0.5 mg/ml penicillin/streptomycin (P/S). Cells lines were grown in a humidified incubator at 37°C in a 5% CO_2_ atmosphere. All cell lines were checked and were mycoplasma-free.

The wild-type cytolethal distending toxin from *E. coli* (CDT Ec) or *H. ducreyi* (CDT Hd) and catalytic dead mutants (CDT^H153A^ and CDT^D273R^, respectively) were produced and purified in the lab at 25 μg/ml (Fedor et al., 2013; Pons et al., 2019) and preserved in phosphate-buffered saline (PBS) (Sigma-Aldrich) with 10% glycerol.

When needed, HeLa cells were treated with ATR inhibitor (ATRi) (VE-821, Sigma-Aldrich, 5 μM).

Quiescence of RKO was induced by cultivation of cells until confluence followed by serum starvation for 2 days. The quiescent cells were treated or not with CDT for 7 h before γH2AX staining.

### Human Samples

Biological samples were obtained from seven different patients treated at the Toulouse University Hospital. Patients gave informed consent and were included in the registered BioDIGE protocol approved by the ethics committee “comité de protection des personnes du Sud-ouest et Outre-mer II, agence régionale de Santé Midi-Pyrénées” and was financially supported by the Toulouse University Hospital (NCT 02874365). Colonic samples were obtained from biopsies of healthy patients undergoing endoscopy.

### DNA Fiber Assay

HeLa or U2OS cells were treated with CDT for 16 or 24 h, respectively, before sequential pulse labeling with 50 μM CldU (5-chlorodeoxyuridine, Sigma-Aldrich), then 100 μM IdU (5 iododeoxyuridine, Sigma-Aldrich) for 20 min each, followed by a chase with 200 μM thymidine (Sigma-Aldrich) for 1 h. Cells were then collected and DNA fiber assays were performed as described previously (Fernandez-Vidal et al., 2019). IdU and CldU were detected with monoclonal mouse (1/50, BD347583, Becton Dickinson) and rat anti-BrdU antibodies (1/75, OBT0030G, Bio-Rad), respectively, and subsequently single-strand DNA with mouse antibody (1/50, MAB3034, Millipore). Images were analyzed using NIS Elements-AR Nikon software. The specific DNA staining allowed the exclusion of any signal due to broken or overlapping DNA fibers. IdU track length was determined if flanked by a CldU track. At least 400 fibers per condition were measured.

### Cell Cycle Analysis by Flow Cytometry

Sixteen hours after CDT treatment (2.5 ng/ml), HeLa and U2OS cells were incubated with 5-ethynyl-2′-deoxyuridine (EdU; 5 μM) for 30 min. Cells were collected by trypsinization and fixed, and incorporated EdU was detected using the baseclick EdU flow cytometry kit (Sigma, BCK-FC488) according to the manufacturer’s instructions. Cells were incubated in PBS containing DAPI (1 μg/ml) for 15 min before samples were processed using flow cytometry (Beckman Coulter CytoFLEX S). At least 10,000 events were analyzed per sample using the CytExpert software.

### Metaphase Spreading and Fluorescence *in situ* Hybridization Analysis

U2OS cells were treated with CDT from *E. coli* at 250 pg/ml during 48 h before adding nocodazole (0.1 μM) for 5 h more. After mitotic shake off, the cells were resuspended in a hypotonic solution (75 mM KCl) and incubated for 20 min at 37°C. Then, the cells were fixed in a methanol/acetic acid solution (3:1) and dropped on slides to spread the chromosomes. The RP11-36B6 and RP11-281J9 BAC probes (mapped onto FRA7H and FRA16D loci, respectively) were labeled by nick translation according to the supplier’s recommendations (VY Nick Translation Kit and VY green dUTP, Abbott Molecular), then precipitated with ethanol (70%), human Cot-1 DNA (0.1 μg/μl, Invitrogen), DNA MB grade (1 mg/ml, Roche), and ammonium acetate (0.3 M) overnight at −20°C. After washing with 70% ethanol, the precipitated DNA was incubated for 15 min at 37°C in hybridization mix composed of 50% formamide, 2X SSC, 10% dextran sulfate, and 1% Tween20 and stored at −20°C. Metaphase slides were incubated at 62°C for 1 h then in 4% formol during 5 min, washed with PBS, followed by dehydration process in successive ethanol baths (70, 80, 90, and 100%) for 1 min each. The probe was applied on metaphases, denatured for 5 min at 80°C, and hybridized overnight at 37°C. Finally, the chromosomes were stained with DAPI (2 μM, 10 min) before adding VECTASHIELD mounting medium (Vector Laboratories). Image acquisition of multiple random fields was performed on a wide-field microscope (model Nikon, Ci-S, × 60 objective).

### EdU Staining and Immunofluorescence Analysis

Cells were grown on glass coverslips. After 23 h of CDT treatment, EdU (10 μM) wad added for 45 min. Then, cells were fixed with 4% paraformaldehyde for 15 min and permeabilized with 0.5% Triton X-100 for 20 min. Incorporated EdU was detected using the baseclick EdU kit (BCK-EdU488, Sigma-Aldrich) according to the manufacturer’s instructions. Then, cells were blocked in 3% bovine serum albumin (BSA) and stained with primary antibodies in a blocking solution. For replication protein A (RPA) detection, a pre-extraction step (0.5% Triton X-100 for 5 min) was performed before fixation. Cyclin A (H3, Santa Cruz, sc-271645, 1/100) antibody was incubated overnight at 4°C, while RPA (Calbiochem, Ab-2, Mouse mAb, RPA34-19, NA18, 1/200) and RIF1 (Bethyl A300-568A-4, 1/1000) antibodies were incubated for 3 h at room temperature. Cells were washed three times with PBS 0.1% Tween20 and incubated with the secondary antibodies (dilution 1/1,000) for 2 h (AlexaFluor purchased from Invitrogen). DNA was stained with DAPI.

For γH2AX immunofluorescence, quiescent or proliferating RKO cells were treated with CDT for 7 h, then fixed and permeabilized with 4% paraformaldehyde and 0.1% Triton X-100 for 15 min, blocked in 1% BSA and 0.1% Triton X-100 for 1 h, and finally stained with γH2AX antibody (Merck/Millipore, 05–636, 1/400) in 1% BSA for 3 h. High-capacity acquisition of fluorescent cell images was obtained by using an ArrayScan HCS with a × 20 objective lens reader, and image analysis was carried out by using the Cellomics analysis software (Thermo Scientific). Cells were positive for γH2AX when > 4 foci/nuclei were detected. For each analysis, a minimum of 1,000 cells were analyzed in three independent experiments. Cell cycle position was determined by quantification of DAPI signal intensity using R software.

### Organoid Culture, Treatment, and Immunofluorescence

Colorectal crypt isolation was performed as described previously (Sébert et al., 2018). Fresh Matrigel (Corning, 356255) was added to isolated crypts; 25 μl of Matrigel containing 50 crypts were plated in each well of a pre-warmed eight-well chamber (Ibidi, 80841). Once the Matrigel had polymerized for 20 min at 37°C, 250 μl of culture medium was added to each well as described previously (Sébert et al., 2018). Then, colorectal crypts were treated or not (NT) with wild-type (25 and 2.5 ng/ml) or mutated (H153A, 25 ng/ml) CDT from *E. coli* and incubated in a humidified incubator at 37°C and 5% CO_2_ for 16 h (day 0). Finally, CDT was removed (day 1), and the culture medium was changed every 3 days without N-acetylcysteine (NAC; Sigma, A9165-5G) and LY2157599 (Axon MedChem, 1941). At day 5, nicotinamide (Sigma, N0636), SB202190 (Sigma, 57067), and PGE2 (Sigma, P0409) were removed from the medium and Wnt3a-conditioned medium [supernatants from L Wnt-3A cells (ATCC^®^ CRL-2647^TM^)] reduced to 5%. At day 6, Wnt3a-conditioned medium was totally removed until day 8.

All cultures were stopped at day 4 or 8 for analysis. EdU (10 μM) was added to the culture medium, 16 h before organoid fixation with 2% of paraformaldehyde for 30 min at 37°C. Then, organoids were washed in PBS and permeabilized with 0.5% Triton X-100 for 40 min. Incorporated EdU was detected using the baseclick EdU kit (BCK-EdU488, Sigma-Aldrich) according to the manufacturer’s instructions. Then, cells were blocked in 3% BSA and stained with γH2AX antibody (1/1,000) in a blocking solution overnight at 4°C. Organoids were washed three times in PBS and incubated with the secondary antibody (1/1,000) for 2 h (AlexaFluor purchased from Invitrogen). After washes, DNA was stained with DAPI (2 μg/ml) for 30 min. Finally, plates were mounted with VECTASHIELD mounting medium.

In order to measure the organoid size, image acquisition of organoids was performed on a bright-field microscope (×5 objective). All organoids present in wells were counted. For immunostaining, at least six random organoids were analyzed for each condition with an inverted confocal microscope (Leica SP8, × 40 objective). Images were analyzed using the ImageJ software from FiJi.

### Statistical Analysis

The results are expressed as the mean ± SEM. Statistical analysis was assessed using Prism 9 software (GraphPad). Student’s *t*-test, Mann–Whitney, and one-way or two-way ANOVA tests, followed by *post hoc* tests were used when appropriate. A *p* value <0.05 was considered significant. For DNA fiber assays, statistical analysis was performed using two-tailed Mann–Whitney test. For cell cycle and interphase bridge analysis, one-way ANOVA followed by Tukey’s multiple comparison test was used. For fragile site expression, chromosomic aberration, and mitotic bridge analysis, Student’s *t*-test was employed. For analysis of cells with DNA bridges after CDT and ATRi treatments, two-way ANOVA followed by Bonferroni’s multiple comparison test was used in order to compare ATRi treatment effects at each dose of CDT exposure. Two-way ANOVA followed by Dunnett’s multiple comparison test was used to study CDT dose effects in the absence or in the presence of ATRi treatment. Two-way ANOVA followed by Tukey’s multiple comparison test was used for cell cycle analysis on cells linked or not with a bridge. To analyze RIF1-cyclin A immunofluorescence in cells linked or not by DNA thread, two-way ANOVA followed by Dunnett’s and Sidak’s multiple comparison tests was used. For γ-H2AX foci formation assays, two-way ANOVA followed by Sidak’s multiple comparison test was performed. For organoid and nucleus size analysis and EdU-positive cell quantification, two-way ANOVA followed by Dunnett’s and Sidak’s multiple comparison tests was used, whereas one-way ANOVA followed by Dunnett’s multiple comparison test was employed for γ-H2AX-positive cell quantification in proliferating cells (EdU plus).

## Results

### CDT Intoxication Induces Replicative Stress in Human Cells

**FIGURE 1:**
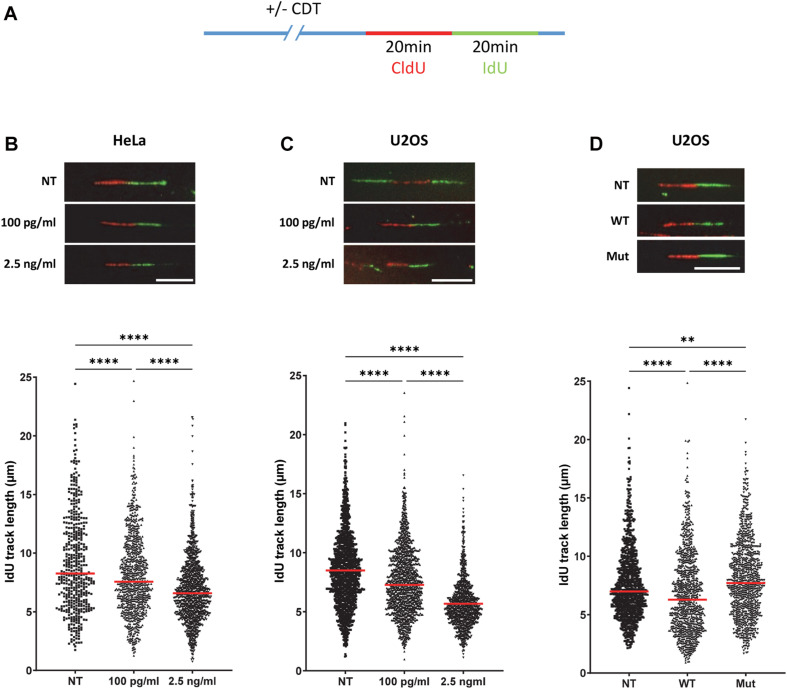
The catalytic activity of cytolethal distending toxin (CDT) induces a replicative stress. **(A)** Cells were incubated or not with CDT holotoxins during several hours before successive pulse labeling with 5-chlorodeoxyuridine (CldU) and 5 iododeoxyuridine (IdU). Then, replication fork speed was analyzed by DNA fiber assay. IdU track length was determined. **(B–D)** Upper panel: representative images of replication tracks: CldU (red) and IdU (green) (*n* > 400 IdU tracks were measured with a wide-field fluorescent microscope, original magnification × 40, scale bar: 10 μm). Lower panel: horizontal red lines represent the median (**P < 0.01, ****P < 0.0001). **(B)** HeLa cells were treated or not (NT) with 100 pg/ml or 2.5 ng/ml of CDT from *E. coli* for 16 h before IdU and CldU staining. **(C)** U2OS cells were treated or not with the same doses of CDT from *H. ducreyi* for 24 h before the replication staining. **(D)** U2OS cells were treated or not with 2.5 ng/ml of wild-type (WT) or catalytically inactive mutant (Mut) of CDT from *H. ducreyi* for 24 h before IdU and CldU incorporation.

In order to investigate the mechanism by which CDT promotes genetic instability, we assessed the toxin impact on the DNA replication program, at a single-molecule level. To address this question, we performed DNA fiber assays and monitored the replication fork velocity. HeLa and U2OS cells were incubated with CDT holotoxins from *E. coli* or *H. ducreyi*, respectively. Then, the successive double-pulse labeling with two nucleotide analogs, CldU followed by IdU incorporation, was performed and IdU track lengths measured (Figure 1A). We observed a significant decrease in IdU track length, revealing a slowing down of replication fork speed in the presence of CDT compared to untreated cells, independently of CDT-producing strains and host cells (Figures 1B,C). The same experiment was performed in U2OS cells with a mutant CDT from *H. ducreyi* in which aspartic acid 273, essential for CdtB catalytic activity, is replaced by an arginine (CDT^D273R^) (Guerra et al., 2005; Pons et al., 2019). U2OS cells cultivated in the presence of CDT^D273R^ displayed a fork speed close to that observed in untreated cells, revealing that CDT catalytic activity is crucial to mediate the slowing down of fork progression (Figure 1D). Altogether, these results demonstrate that CDT holotoxins induce a replicative stress in different host cells and underline the major role of CDT catalytic activity in this process.

**FIGURE 2:**
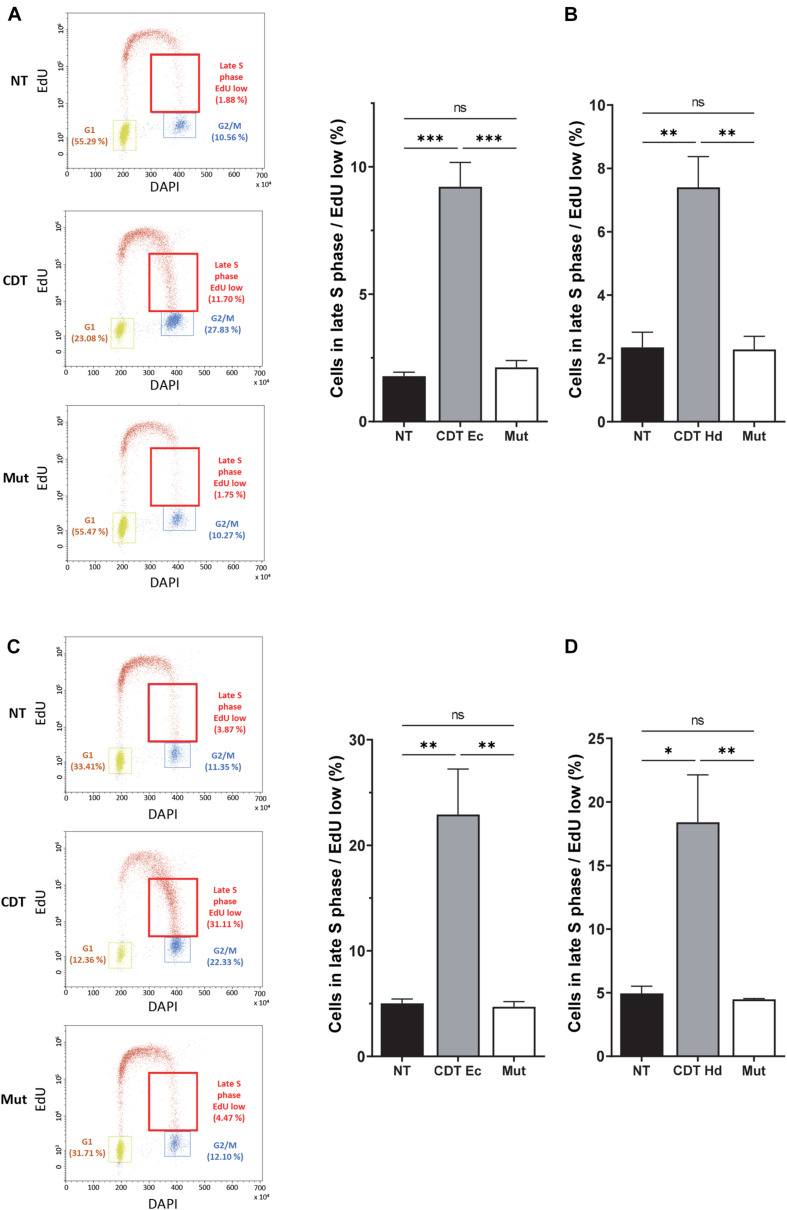
CDT causes a slowdown of DNA replication in the late S phase. Cell cycle analysis by flow cytometry of HeLa **(A,B)** and U2OS **(C,D)** cells treated or not with 2.5 ng/ml of wild-type or catalytically inactive CDT mutant (Mut) from *E. coli* (CDT Ec) **(A,C)** or *H. ducreyi* (CDT Hd) **(B,D)** for 16 h was performed. **(A,C)** Left panel: representative flow cytometry of cells treated with CDT Ec is shown. EdU incorporation is plotted against the cellular DNA content (DAPI). Quantification of G1, G2, and late S cell population with low EdU (red boxes) is indicated. Right panel: data represent the percentage of EdU weakly positive cells in the late S phase. At least 10,000 events were analyzed per sample using the CytExpert software (mean ± SEM of at least three independent experiments) (^∗^*P* < 0.05, ^∗∗^*P* < 0.01, ^∗∗∗^*P* < 0.001, and ns, not significant).

We next analyzed the consequences of this replicative stress on the global cell distribution in the S phase by performing EdU incorporation experiments followed by flow cytometry analysis. In addition to the G2/M block, the examination of EdU incorporation according to DAPI staining showed a higher proportion of S phase cells (EdU-positive cells) at the border of G2/M after *E. coli* or *H. ducreyi* wild-type CDT exposure of HeLa cells compared to control cells (Figure 2A, red boxes and Figure 2B). Indeed, CDT treatment generated a significant increase in the proportion of cells in the late S phase with a low EdU incorporation, which is abolished with the mutant form. Very similar results were obtained in U2OS cells (Figures 2C,D). These experiments unveil a slowing down of DNA replication occurring probably mainly in the late S phase or a weak replication persisting in G2, after CDT intoxication. Despite the wild-type p53 status, a G1 block has not been detected in U2OS cells, in agreement with previous a work (Blazkova et al., 2010).

### CDT Exposure Promotes Mitotic Abnormalities and Fragile Site Expression

**FIGURE 3:**
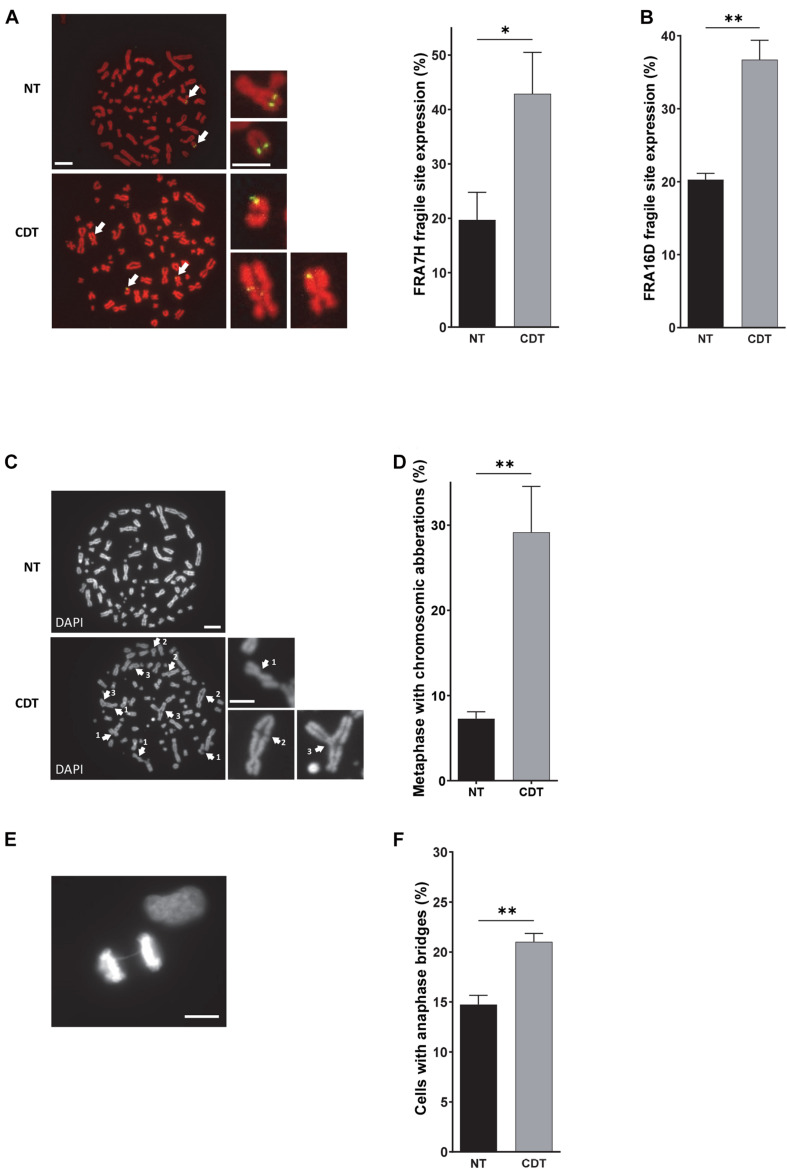
CDT induces the expression of fragile sites, global chromosomic aberrations, and anaphase bridges. **(A)** Illustration and quantification of metaphases with the expression of the common fragile site FRA7F (green) analyzed by fluorescence *in situ* hybridization (FISH) in U2OS cells treated or not with 250 pg/ml of CDT from *E. coli* (CDT). The chromosomes were stained with DAPI (red). Images were obtained with a wide-field fluorescent microscope. *N* > 30 metaphases, scale bar: 10 μm (5 μm, chromosome magnification). **(B)** Quantification of metaphases with the expression of the common fragile site FRA16D analyzed by FISH in U2OS cells treated or not (NT) with *E. coli* CDT (CDT) for 53 h. Illustration **(C)** and quantification **(D)** of metaphases containing at least one chromosomic aberration in U2OS cells treated or not (NT) with 250 pg/ml of CDT from *E. coli* (CDT) during 53 h. The chromosomes were stained with DAPI (grayscale). Images were obtained with a wide-field fluorescent microscope. *N* > 60 metaphases; white arrows indicate chromosomal abnormalities such as DNA break (1), end-to-end fusion (2), and triradial chromosomes (3). Scale bar: 10 μm (5 μm, chromosome magnification). Representative image of DAPI (grayscale) staining **(E)** and quantification **(F)** of anaphases with DNA bridge in HeLa cells treated or not (NT) with 100 pg/ml of CDT from *E. coli* (CDT) for 24 h. *N* > 90 anaphases were analyzed with a wide-field fluorescent microscope. Scale bar: 10 μm (mean ± SEM of at least three independent experiments) [^∗^*P* < 0.05, ^∗∗^*P* < 0.01, *versus* non-treated (NT)].

Among the domains replicated in the late S phase, common fragile sites (CFS) constitute the major chromosomal regions prone to breakage upon moderate replicative stress and the main source of genomic instability in precancerous lesions and cancer development (Gorgoulis et al., 2005; Durkin and Glover, 2007; Bignell et al., 2010; Georgakilas et al., 2014). Therefore, we explored CFS stability by using a FISH (fluorescence *in situ* hybridization)-based assay after cell treatment with CDT. We quantified the percentage of cells with rearrangements (translocation, amplification, or deletion) that localized to the FRA7H and FRA16D fragile sites. For that, we used U2OS cell line in which these fragile sites are not already rearranged. As shown in Figures 3A,B, we highlighted a significant increase in the expression of both fragile sites in cells exposed to the genotoxin compared to control cells, supporting that the replicative stress induced by CDT may contribute to the establishment of genomic instability by at least expression of fragile sites.

Afterward, we investigated the consequences of CDT exposure on the global chromosomal integrity. Metaphase spreads revealed that after CDT intoxication, the U2OS cell proportion with structural abnormalities significantly increased compared to control cells (Figures 3C,D). Chromatid breaks, end-to-end fusions, and radial chromosomes were observed (see Figure 3C for examples), depicting a huge chromosomal instability induced by the toxin. Then, we monitored chromosome segregation in anaphase and highlighted a significant increase of cells with persistent physical connections between the two DNA batches called DNA bridges after CDT treatment compared to control cells (Figures 3E,F and Supplementary Figure 1). These results suggest that the chromosomal abnormalities induced by CDT impair proper chromosome segregation in anaphase.

### Genetic Instability Driven by CDT Is Transmitted to Daughter Cells

**FIGURE 4:**
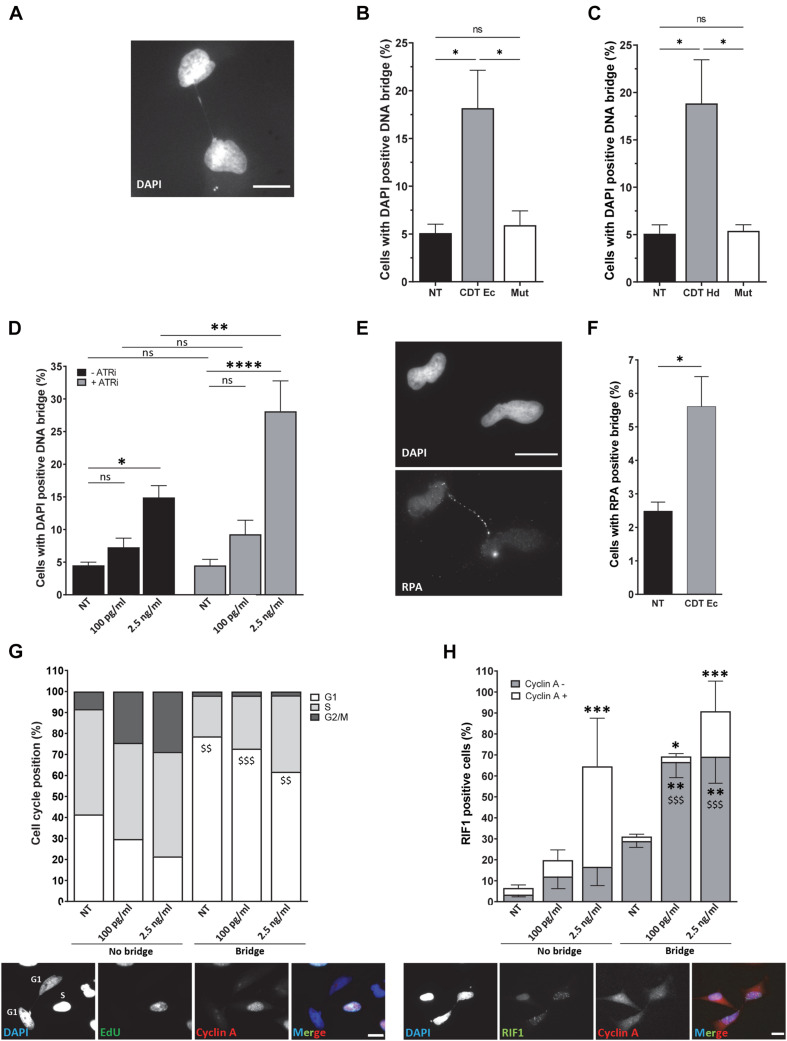
ATR prevents the formation of persistent DNA bridges between G1 daughter cells. Illustration **(A)** and quantification **(B)** of HeLa cells with DAPI-positive DNA bridge (grayscale) after treatment or not (NT) with 2.5 ng/ml of wild-type (CDT Ec) or catalytically inactive CDT mutant (Mut) from *E. coli* for 24 h. *N* > 500 cells were analyzed with a wide-field fluorescent microscope. Scale bar: 20 μm. **(C)** Quantification of HeLa cells with DAPI-positive DNA bridge after treatment or no treatment (NT) with 2.5 ng/ml of wild-type (CDT Hd) or catalytically inactive CDT mutant (Mut) from *H. ducreyi* for 24 h. *N* > 500 cells were analyzed with a wide-field fluorescent microscope (mean ± SEM of four independent experiments) (^∗^*P* < 0.05, ns, not significant). **(D)** Quantification of HeLa cells with DAPI-positive DNA bridge after treatment or non-treatment (NT) with 100 pg/ml or 2.5 ng/ml of CDT from *E. coli* and ATR inhibitor (ATRi) for 24 h. *N* > 500 cells were analyzed with a wide-field fluorescent microscope (mean ± SEM of four independent experiments) (^∗^*P* < 0.05, ^∗∗^*P* < 0.01, ^****^P < 0.0001; ns, not significant). **(E)** Representative images of replication protein A (RPA) immunostaining (grayscale) and quantification **(F)** of HeLa cells with RPA-positive DNA bridge after treatment or no treatment (NT) with 2.5 ng/ml of CDT from *E. coli* (CDT Ec) for 24 h. Nuclei were stained with DAPI (grayscale) and *n* > 500 cells were analyzed with a wide-field fluorescent microscope. Scale bar: 20 μm (mean ± SEM of four independent experiments) [^∗^*P* < 0.05, *versus* non-treated (NT)]. **(G)** Upper panel: quantification of cell cycle position of HeLa cells linked or not with a bridge after *E. coli* CDT treatment [100 pg/ml or 2.5 ng/ml or non-treated (NT)] for 24 h (mean ± SEM of three independent experiments). Cyclin A and EdU-negative cells are counted in G1, cyclin A, and EdU-positive cells in S and cyclin A-positive and EdU-negative cells in G2. Lower panel: representative images of EdU (grayscale) and cyclin A (grayscale) immunostaining of HeLa cells treated with CDT from *E. coli* (2.5 ng/ml) for 24 h. Nuclei were stained with DAPI (grayscale). Merge was performed with DAPI (blue), EdU (green), and cyclin A (red) images. *N* > 100 cells were analyzed with a wide-field fluorescent microscope. Scale bar: 20 μm (^$$^*P* < 0.01, ^$$$^*P* < 0.001 *versus* no bridge at the same CDT dose for the G1 phase). **(H)** Upper panel: quantification of positive HeLa cells for RIF1 ± cyclin A staining after *E. coli* CDT treatment [100 pg/ml or 2.5 ng/ml or non-treated (NT)] for 24 h (mean ± SEM of three independent experiments) [^∗^*P* < 0.05, ^∗∗^*P* < 0.01, ^∗∗∗^*P* < 0.001 *versus* non-treated (NT) and ^$$$^*P* < 0.001 *versus* no bridge at the same CDT dose]. Lower panel: representative images of RIF1 (grayscale) and cyclin A (grayscale) immunostaining of HeLa cells treated with CDT from *E. coli* (2.5 ng/ml) for 24 h. Nuclei were stained with DAPI (grayscale). Merge was performed with DAPI (blue), RIF1 (green), and cyclin A (red) images. *N* > 100 cells were analyzed with a wide-field fluorescent microscope. Scale bar: 20 μm.

To deeper understand the fate of cells presenting these mitotic defects, we monitored chromatin abnormalities in interphase. Strikingly, nuclei connected with a thin chromatin bridge stained with DAPI appeared more frequently after exposure to wild-type CDT from *E. coli* or *H. ducreyi* than in untreated HeLa cells or cells treated with the catalytic dead CDT mutant (Figures 4A–C). These persisting DNA double-stranded structures (DAPI-positive bridges) present in interphase between daughter cells could reflect a failure of anaphase bridge resolution during mitosis and transmitted to the next generation. To evaluate whether the replicative stress induced by CDT exposure could be involved in the formation of DNA bridges observed in interphase, we quantified the percentage of cells in interphase presenting DNA bridge after or no treatment with an ATR inhibitor (ATRi). First, we confirmed that wild-type CDT, but not the catalytically inactive CDT mutant, induces the phosphorylation of replication protein A (RPA) on serine 33, an ATR-specific target. RPA phosphorylation was also impaired after ATRi treatment (Supplementary Figure 2). Furthermore, as shown in Figure 4D, ATRi alone did not induce DNA bridges. However, the co-treatment with CDT and ATRi significantly increase the percentage of cells connected with a DAPI-positive DNA bridge compared to CDT-intoxicated cells without ATRi (14.9% *versus* 28.1% for the CDT highest dose). These data support the major role of ATR to limit the formation of aberrant chromatin structure between daughter cells induced by CDT and sustain the involvement of replicative stress to drive genetic instability.

To further characterize the nature of DNA bridges formed after CDT intoxication, we asked whether single-stranded structures may link nuclei of daughter cells. To this end, we monitored the recruitment on the bridge of RPA, a protein known to cover and protect single-stranded DNA (Figures 4E,F). We found that CDT exposure stimulates the formation of RPA-positive bridges connecting the nuclei of daughter cells compared to untreated cells (2.5 *versus* 5.6%) but likely at a lesser extent compared to double-stranded bridges (compare Figures 4B,F). Altogether, these results demonstrate that single- and double-stranded DNA bridges connecting the nuclei of daughter cells increase after CDT intoxication, suggesting the transmission of aberrant chromatin structures to the next cell generation.

Then, we analyzed the impact of interphase bridges on cell cycle progression. To address this question, we performed EdU incorporation to track cells in the S phase, together with cyclin A immunostaining to monitor cells in the G1 phase (cyclin A-negative cells) (Figure 4G). We show that without CDT treatment, cells connected with a chromatin bridge are mainly in the G1 phase of the cell cycle (78.6%). In agreement with previously reported cell cycle arrest, CDT exposure seems to induce an accumulation of cells not linked with a DNA thread in the G2 phase (8.4% of untreated cells are in G2 *versus* 28.7% after treatment with 2.5 ng/ml of CDT) correlated with a decrease of G1 phase (41.5% of untreated cells are in G1 *versus* 21.5% after treatment with 2.5 ng/ml of CDT) (Figures 2A, 4G). However, cells connected with a bridge are mainly in the G1 phase with a slight but not significant increase in the S phase after CDT treatment (Figure 4G). These data suggest that CDT intoxication promotes the emergence of DNA thread between cells mostly in the G1 phase of the cell cycle, probably until their resolution before or within the next S phase.

To determine whether replicative stress could be the cause of G1 cells connected with a thin DNA thread, we monitored Rap1 interacting factor 1 (RIF1) foci formation. RIF1 constitutes a major factor playing a crucial role in genome maintenance after replicative stress. Indeed, RIF1 is associated with stalled DNA replication forks favoring their restart (Buonomo et al., 2009; Alabert et al., 2014; Garzón et al., 2019; Mukherjee et al., 2019). Moreover, RIF1 was recently found to carry out its activity during and after a perturbed S phase to protect against replicative stress throughout the cell cycle and to ensure chromosome integrity (Harrigan et al., 2011; Lukas et al., 2011; Moreno et al., 2016; Watts et al., 2020). Whatever the presence or absence of chromatin bridges, CDT intoxication induces a significant increase in the percentage of RIF1-positive cells, compared to untreated cells (from 6.5 to 64.5% RIF1-positive cells among the cells not linked with a bridge and from 31.1 to 90.8% RIF1-positive cells among those connected with a DNA bridge) (Figure 4H). Moreover, a significant increase of RIF1-positive cells was observed in G1 (cyclin A-negative cells) for cells linked with DNA thread, in contrast to cells without DNA bridge. Indeed, among cells connected with a DNA bridge, 28.9% are RIF1 positive in the G1 phase in untreated condition compared to 69.2% after CDT exposure to 2.5 ng/ml. Our finding thus indicates that CDT treatment causes a massive RIF1 recruitment, not only in the G1 phase in nuclei linked with a DNA bridge but also in S and G2 in cells without an interphase bridge.

### CDT Promotes γH2AX Foci Formation in Cycling Cells

**FIGURE 5:**
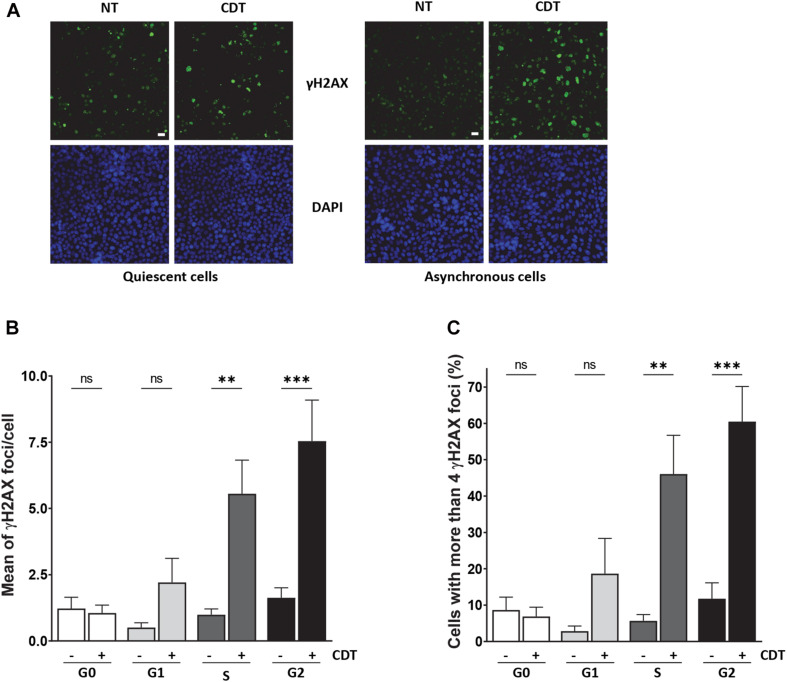
CDT induces γH2AX foci formation preferentially in proliferating cells. **(A)** Representative images of γH2AX (green) immunostaining in quiescent or proliferating RKO cells after exposure or non-exposure (NT) with *E. coli* CDT (25 ng/ml) for 7 h. DNA was stained with DAPI (blue). Scale bar: 20 μm. **(B)** Quantification by ArrayScan analysis of γ-H2AX foci formation in quiescent or proliferating RKO cells sorted according to cell cycle phase after CDT treatment from *E. coli* (25 ng/ml) for 7 h. **(C)** Quantification of γH2AX-positive cells with > 4 foci/nuclei in cells treated as in **(B)**. For each analysis, a minimum of 1,000 cells were analyzed with a wide-field fluorescent microscope (ArrayScan technology) in three independent experiments [^∗∗^*P* < 0.01, ^∗∗∗^*P* < 0.001 *versus* non-treated (NT); ns, not significant].

As we observed that CDT intoxication leads to replicative stress, we hypothesized that proliferating cells could be more sensitive to the toxin compared to quiescent cells. To address this question, quiescence was induced in RKO human colonic cells by confluence and serum starvation (Supplementary Figure 3). Then, cycling or quiescent cells were incubated with CDT holotoxin from *E. coli*, and DNA damage induction was measured through γH2AX immunostaining and analyzed by the ArrayScan technology (Figure 5A). In cycling cells, cell cycle distribution was established according to DAPI signal intensity. In quiescent cells (G0) exposed to CDT, no significant variation of γH2AX foci number per cell or in the proportion of γH2AX-positive cells (with more than four foci) was observed (Figures 5B,C). However, CDT-intoxicated cycling cells presented more γH2AX foci per cell, and the percentage of γH2AX-positive cells significantly increased compared to untreated cells, especially in the S and G2 phases (Figures 5B,C). These data are consistent with the induction of replicative stress (Figure 1) and support the notion that proliferating cells could be more sensitive than quiescent cells to the genotoxicity induced by CDT.

**FIGURE 6:**
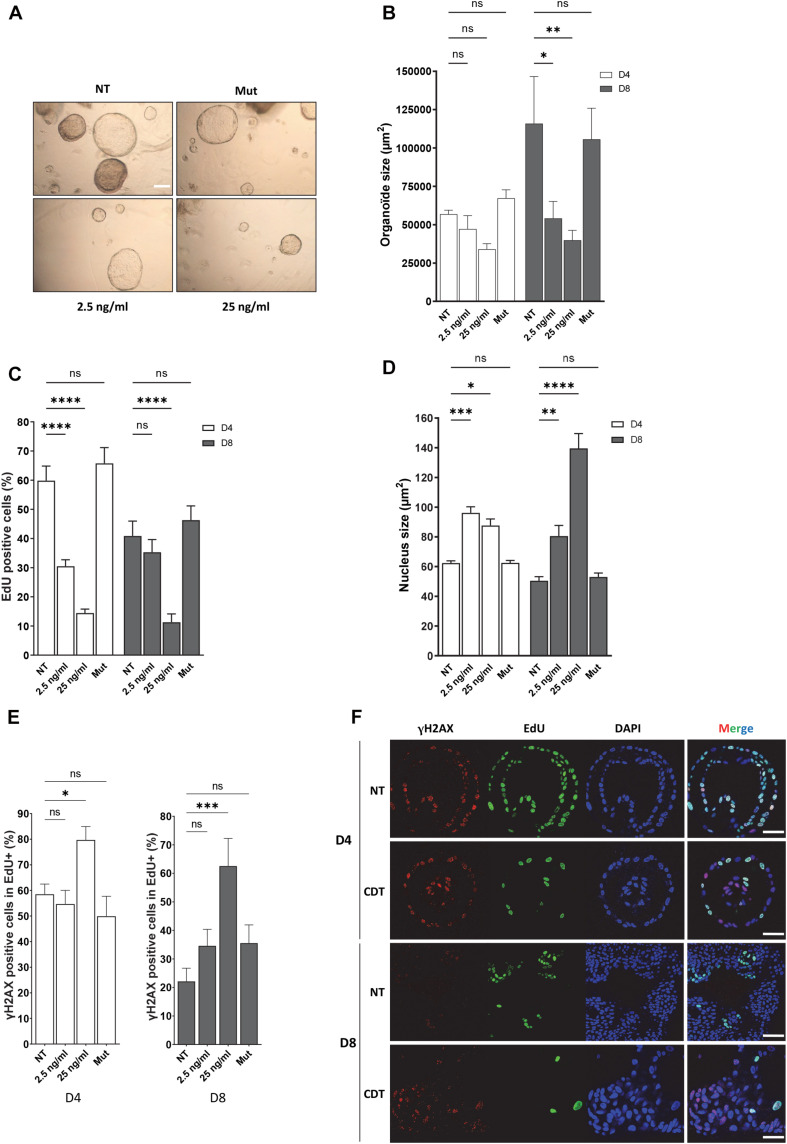
CDT causes nuclear distension associated with DNA damage in cycling cells of human colorectal organoids leading to decrease of growth. Human colorectal crypts were treated at day 0 with wild-type (2.5 and 25 ng/ml) or catalytic inactive mutant (H153A, 25 ng/ml, Mut) of CDT from *E. coli* for 16 h. **(A)** Representative images of organoids at day 8 with a bright-field microscope. Scale bar: 200 μm. **(B)** At days 4 and 8 of culture, organoid size was measured. *N* > 10 organoids. **(C)** EdU was added to the organoid culture medium for 16 h before fixation. Then, EdU was revealed and EdU-positive cells per organoid were quantified by confocal analysis. *N* > 6 organoids. **(D)** At days 4 (D4) and 8 (D8) of culture, organoid nucleus size was analyzed by confocal microscopy. *N* > 6 organoids. **(E,F)** At day 4 (D4) or 8 (D8), EdU was added to the organoid culture medium for 16 h before fixation and revealed. Then, γH2AX immunostaining was performed and γH2AX-positive cells in proliferating cell population (EdU+) from organoids were quantified. *N* > 6 organoids. **(F)** Representative images of EdU (green) and γH2AX (red) immunostaining in human colorectal organoids at days 4 (D4) and 8 (D8) treated or non-treated (NT) with 25 ng/ml of *E. coli* CDT were obtained from confocal analysis. Scale bar: 50 μm (mean ± SEM of at least three independent experiments) [^∗^*P* < 0.05, ^∗∗^*P* < 0.01, ^∗∗∗^*P* < 0.001, ^****^P < 0.0001 *versus* non-treated (NT); ns, not significant].

To reinforce this finding in a more physiological model, we used human organoids in culture. As CDT-producing *E. coli* were associated with colorectal cancer (Buc et al., 2013), we performed human colorectal organoid culture in the presence of CDT holotoxin from *E. coli.* Human colon crypts were purified from fresh biopsies from healthy donors. In order to get as close as possible to the physiological context, isolated crypts were seeded in 3D Matrigel and directly incubated with the CDT holotoxin from *E. coli* during 16 h, time required for the opened crypts to seal and form cysts. Then, the free toxin was removed from the medium but not the one trapped in the cyst, and the organoid growth was monitored during 8 days. This protocol has the advantage of exposing fresh crypts to CDT and assessing the toxin impact on the organoid growth during several days without passaging and therefore maintaining their integrity. Figures 6A,B illustrates that colorectal organoid size was significantly smaller after 8 days of CDT exposure compared to untreated organoids. Organoids were also incubated with a mutated CDT (CDT^H153A^), in which histidine 153, a crucial residue for the catalytic activity of CdtB, was replaced by an alanine (Elwell and Dreyfus, 2000). The organoid size was not significantly affected by exposure to the catalytic inactive CDT mutant. Then, we asked whether the CDT-induced organoid growth defect could be due to a slowing down of cell proliferation. To achieve this, we monitored proliferating cells with EdU incorporation after CDT intoxication of colorectal organoids (Figure 6C). First, as expected, we observed a significant decrease in the EdU-positive cell proportion from day 4 to day 8 without any treatment, confirming a decrease of cell proliferation during the organoid differentiation process. Moreover, exposure to wild-type CDT generated a huge decrease of cell proliferation at day 4, which was maintained until day 8. In contrast, treatment with CDT-catalytic-dead mutant did not significantly alter cell proliferation. Collectively, these data indicate that CDT affects cell proliferation through its catalytic activity, impairing organoid growth. Furthermore, as shown in Figure 6D, nucleus size increased from day 4 at both doses of CDT and became much larger on day 8 after CDT treatment at 25 ng/ml, highlighting a nuclear distension in human colorectal organoids, characteristic of CDT intoxication. However, we did not observe any significant variation after intoxication with the catalytic inactive mutant of CDT underlining the importance of its catalytic activity in this process. As CDT induces replicative stress (Figure 1), we finally wondered whether CDT intoxication could generate γH2AX accumulation in proliferating cells. For this purpose, we analyzed the proportion of γH2AX-positive cells in the EdU-positive cell population (Figures 6E,F). Interestingly, these experiments revealed that 4 days after toxin exposure with 25 ng/ml of CDT, the proportion of γH2AX-positive cells in proliferating cells (EdU+) was higher in intoxicated organoids compared to controls. In addition, 8 days after CDT intoxication, this increase was maintained in the cells, keeping their proliferation status. Finally, no significant variation was observed after intoxication of organoids with the CDT catalytic inactive mutant, indicating that γH2AX accumulation in proliferating cells of human colorectal organoids is dependent on its catalytic activity. Altogether, our results indicate that CDT induces γH2AX accumulation in proliferating cell population persisting through human colorectal organoid differentiation (day 8).

## Discussion

Cytolethal distending toxin produced by several bacterial strains was reported to promote not only cancer hallmark acquisition in chronically intoxicated cells but also tumorigenesis in mice models (Ge et al., 2007, 2017; Guidi et al., 2013; Graillot et al., 2016; He et al., 2019). Although some evidences support that CDT generates DNA breaks associated with genetic instability such as mutagenesis, chromatin and chromosomal abnormalities, and micronucleus formation (Frisan et al., 2003; Fedor et al., 2013; Guidi et al., 2013), the mechanism leading to cancer development is still unclear. Indeed, data are still lacking to explain how DNA breaks drive genetic instability transmitted to the next generation and to characterize CDT cellular targets allowing a better understanding of CDT *in vivo* tumorigenic properties. For this, mechanism-based approaches were led not only in HeLa cells, widely used for CDT studies, but also in U2OS cells, two well-characterized cellular models. In addition, RKO colorectal cells and human colorectal organoids were used to investigate the physiological impact of CDT. Here, we report that CDT exposure leads to a replicative stress associated with mitotic aberrations and persistent chromatin abnormalities connecting daughter cells in G1. Our data start to fill the knowledge gap by highlighting that the proliferative status of CDT host cells may be crucial and determine the tumor cell fate. To our knowledge, this is the first time that the impact of CDT intoxication is directly addressed in human organoids from healthy donors.

First, we demonstrate that CDT exposure induces a dose-dependent slowing down of replicative fork dynamic in HeLa and U2OS cells. Since these results were independent of the human cell type and CDT-producing strains, and given that the catalytic mutant has no effect on the fork velocity, we can speculate that the replicative stress induced by CDT could be a general mechanism consecutive to DNA breaks. These data reinforce our previous report indicating that CDT-induced SSB are converted into DSB upon DNA replication resulting in S phase delay (Fedor et al., 2013). Moreover, single-stranded DNA coated by RPA obtained after CDT treatment suggests uncoupling between replicative helicase and DNA polymerases (Fedor et al., 2013). Subsequently, ATR-dependent replicative stress response and FA pathway seem to be required to overcome the replication fork stalling induced by CDT (Fahrer et al., 2014; Bezine et al., 2016). Altogether, these evidences hint at a CDT-induced replicative stress that we definitively confirmed with replication fork progression defects on single DNA fibers. Our finding substantiates the importance of cell proliferation for CDT genotoxicity. Other workers have suggested that the S phase could be crucial for CDT intoxication. Indeed, Comayras et al. (1997) showed that most cells exposed to CDT in G2 and M were arrested only at the subsequent late G2 phase, in contrast to cells intoxicated in G1 or S phase, which were blocked in the G2 phase of the current cell cycle (Comayras et al., 1997; Sert et al., 1999). These results support that the passage through the S phase is required for CDT to exert its toxic effect in good agreement with the CDT-mediated replicative stress. Thus, it is reasonable to think that CDT activity may be mainly directed to single-stranded DNA predominantly generated during the DNA replication process. We can speculate that some DNA regions prone to adopt single-stranded DNA structure could also constitute CDT favor substrates. Moreover, since the slowing down of DNA replication occurs mainly in the late S phase after CDT exposure, CFS represent excellent target candidates. Indeed, in addition to their late replication and their high sensitivity to moderate replicative stress, their fragility can be explained by several features such as an enrichment in large genes, poor in DNA replication origins, forming secondary DNA structures due to AT-rich sequences and linked to 3D genome organization (Georgakilas et al., 2014; Sarni et al., 2020). Consequently, we detected a significant increase in FRA7H and FRA16D fragile site expression upon CDT exposure supporting our hypothesis and illustrating the consequences of the replicative stress. Furthermore, CDT-intoxicated cells displayed various chromosomal abnormalities in metaphase, showing that the host cells unsuccessfully repaired some CDT-induced DNA strand breaks. This suggests that CDT could also target DNA regions other than CFS that might be interesting to characterize in order to deepen CDT mode of action.

Interestingly, our finding revealed a higher frequency of anaphase and interphase DNA bridges after acute exposure to the genotoxin. These bridges may arise from end-to-end chromosome fusions after CDT-induced DNA breakage mentioned above or by incomplete DNA replication, as ATRi treatment amplified their occurrence. Since interphase bridges increase after CDT exposure, we can speculate that some anaphase chromosome bridges persist for many hours into the subsequent cell cycle without breaking as sustained by previous studies (Steigemann et al., 2009; Maciejowski et al., 2015; Pampalona et al., 2016; Umbreit et al., 2020). Finally, a recent work indicates that the bridges broke later, requiring actomyosin forces and initiating chromothripsis, which is further amplified through each mitosis leading to frequent mis-segregation (Umbreit et al., 2020). This is in accordance with our data showing that anaphase and interphase bridges appeared 1 or 2 days, respectively, after acute CDT treatment, thus constituting an early process in the genetic instability setting up. Moreover, they seem to be maintained after chronic intoxication to CDT (Guidi et al., 2013). We further showed that cells with a cytoplasmic DNA bridge are preferential in the G1 phase. This finding suggests that inheritance of lesions from previous cell cycle may correlate with a G1 delay in the next one. This data is in agreement with the work of Lezaja and Altmeyer (2018) highlighting the correlation between the amount of replication remnants and the next G1 duration. Therefore, unresolved DNA damage generated by CDT intoxication would be transmitted to the daughter cells and constitute a major source of genomic instability.

Then, we observed that human quiescent cells seem to be less sensitive to *E. coli* CDT exposure than proliferating cells, supporting the requirement of S phase progression for DNA damage expansion and genetic instability setting up. However, previous studies reported that, despite toxicity was dependent on cell differentiation stage, *A. actinomycetemcomitans*, *H. ducreyi*, or *C. jejuni* CDT can also intoxicate non-proliferating monocyte cells such as dendritic cells and macrophages, resulting in apoptosis cell death (Li et al., 2002; Xu et al., 2004; Hickey et al., 2005; Rabin et al., 2009). This suggests that CDT intoxication does not imperatively require replicative stress induction for killing its cell hosts. However, the previous works only focused on the CDT-mediated DNA damage and/or apoptosis but never monitored the fate of the genetic instability mediated by the toxin. To date, the importance of cell proliferation status on CDT toxicity was never addressed in non-hematological cells. Here, we address for the first time this question in colorectal cells. Thus, these discrepancies could be due to cell type specificity. Furthermore, the methodology that we employed constitutes a physiological process to push proliferating cells in quiescence by confluence and serum starvation. This protocol presents the advantage of comparing the same cell type in two different cell cycle stages (G0 phase *versus* cycling cells), without differentiation induction. Nonetheless, the cellular and molecular modifications induced by quiescence may not be comparable to those operating during the proliferation arrest accompanying the differentiation process. Finally, we cannot exclude that higher CDT doses could induce some DNA damage in quiescent cells. However, our data clearly established that cycling cells are far more sensitive.

To deeper understand the CDT mechanism of action in a more complex and physiological model, we used human colorectal organoids. Fresh human colorectal crypts from healthy donors were directly exposed to *E. coli* CDT until its trapping inside the cysts. This model mimics physiological CDT exposure to the crypts and allows us to monitor organoid growth during several days (8 days), without disrupting the structures, and address different questions. Our data substantiate that, in addition to inducing nuclear distension, CDT intoxication strongly affects colorectal organoid growth by reducing the proliferating rate at least up to 8 days. However, the remaining cycling cells displayed a γH2AX increase a few days after CDT exposure, suggesting either the genotoxin is always active and continuously harms DNA or not all DNA lesions are completely repaired, meaning a persistence of DNA damage over time. After 8 days of organoid culture, stem cells and progenitors should constitute remaining cycling cells, suggesting that CDT could target these cell types and induce DNA injuries. Surprisingly, a high basal level of γH2AX was observed in untreated organoids at 4 days of culture, which can be explained by the high rate of proliferating cells (EdU+) owing to the greatly increased number of S phase occurring in these cells to quickly generate a mature organoid structure. Consequently, cycling cells from colorectal organoids could be more vulnerable to DNA damage during replication such as replication fork collapse leading to DNA breaks. Indeed, embryonic stem cells display marks of replicative stress associated with fast proliferation, and then the constitutive DNA damage response activation is rapidly abolished during differentiation (Ahuja et al., 2016). Moreover, the intestinal epithelium renewing supported by the intestinal stem cells is very frequent with a replacement every 4–5 days, revealing a huge proliferation rate to maintain the tissue homeostasis (Vermeulen and Snippert, 2014). Nevertheless, we cannot exclude that crypt isolation followed by the *in vitro* culture also generates cellular stress responsible for γH2AX induction during the first days.

In conclusion, this model highlights that human primary colorectal cells respond to CDT intoxication by a cell cycle arrest induction. However, a weak proportion of persistent cycling cells is present in mature organoids after CDT exposure. Because these cycling cells display more DNA lesions, probably due to their increased proliferation rate, they are likely to transmit DNA defects on the next generation. Unrepaired or incorrectly repaired lesions might then enhance the probability of mutation accrual, affecting genomic stability and promoting tumor initiation. Moreover, inflammatory context such as chronic inflammatory bowel disease may constitute a permissive environment for CDT intoxication predisposing to tumor progression. Finally, this work raises several questions such as the CDT impact on colorectal differentiation process, as well as its effect on epithelial barrier permeability. Further studies will be required to test these hypotheses, answer these questions, and fully understand CDT pathogenicity.

## Data Availability Statement

The original contributions presented in the study are included in the article/Supplementary Material, further inquiries can be directed to the corresponding author/s.

## Ethics Statement

The studies involving human participants were reviewed and approved by the ethics committee “comité de protection des personnes du Sud-ouest et Outre-mer II, agence régionale de Santé Midi-Pyrénées” (NCT 02874365). Written informed consent to participate in this study was provided by the participants’ legal guardian/next of kin.

## Author Contributions

AF-V designed, conceived, and supervised the study. AF-V, WT, EL, FM, and SH performed the experiments, analyzed the data, and prepared draft figures. EM, LA, and DB performed the endoscopies and the biopsies, and AF and MQ provided their expertise on organoid culture. JV purified CDT holotoxins and provided his expertise on CDT. VB provided BAC probes, material, and her expertise for FISH experiment and helped to edit the manuscript. AF-V prepared the manuscript draft with important intellectual input from JV, DT, AF, and GM. All authors approved the final manuscript. GM and AF-V obtained funding.

## Conflict of Interest

The authors declare that the research was conducted in the absence of any commercial or financial relationships that could be construed as a potential conflict of interest.

## Abbreviations

ATM: ataxia–telangiectasia mutated kinase
ATR: ataxia–telangiectasia and Rad-3-related kinase
ATRi: ATR inhibitor
CDT: cytolethal distending toxin
CDT Ec: cytolethal distending toxin from *E. coli*
CDT Hd: cytolethal distending toxin from *H. ducreyi*
CFS: common fragile sites
CldU: 5-chlorodeoxyuridine
DSB: double-strand break
EdU: 5-ethynyl-2 ′ -deoxyuridine
FA: Fanconi anemia
IdU: 5 iododeoxyuridine
NHEJ: non-homologous end joining
RH: homologous recombination
RPA: replication protein A
SSB: single-strand breaks
SSBR: single-strand break repair.
