# Supplementary material for: Cytolethal Distending Toxin Promotes Replicative Stress Leading to Genetic Instability Transmitted to Daughter Cells
Source: Front Cell Dev Biol. 2021 May 7;9:656795. doi: 10.3389/fcell.2021.656795 (PMC8138442; doi:10.3389/fcell.2021.656795)
Supplement: Supplementary file 1 [file Data_Sheet_1.PDF]

Supplementary Material

1 Supplementary Data: Methods for supplementary Figures

Western blot analysis:

Cells were resuspended and incubated in lysis buffer (50 mM Tris HCl pH 7.5, 250 mM NaCl and 0.5% NP40, HaltTM Protease and Phosphatase inhibitor cocktail (Thermo Scientific)) for 30 min on ice, then sonicated and centrifuged. The soluble fraction was kept and the proteins separated by SDS-PAGE then transferred to a nitrocellulose membrane (Amersham). Membranes were saturated with a 1/1 mixture of blocking buffer (Rockland, Immunochemicals Inc.) and TBS-Tween 0.1%, then incubated with the primary antibody for 16 hours. RPA32 antibody (NA18, dilution 1/200) was purchased from Calbiochem and p-RPA32 (A300, dilution 1/1000) was purchased from Bethyl. The secondary anti-mouse or anti-rabbit HRP-conjugated antibodies (Jackson Immunoresearch laboratories) were incubated for 1 hour. Proteins were visualized with the enhanced chemiluminescence substrate ECL (Biorad) and imaged using the ChemiDoc XRS Biorad Imager and Image Lab Software.

Cell cycle analysis by flow cytometry:

To monitor quiescence induction in RKO cell line after confluence and serum starvation, cells were collected by trypsinization, washed, fixed by cold ethanol 75% and store overnight at -20°C. Then, cells were washed with PBS and treated with RNAse A (400µg/ml) and Propidium Iodide (40µg/ml) for 30 min. Finally, samples were processed using flow cytometry (MACSQuant, Miltenyi Biotec). At least 10,000 events were analyzed per sample using FlowJo software.

2 Supplementary Figures and Tables

2.1 Supplementary Figure

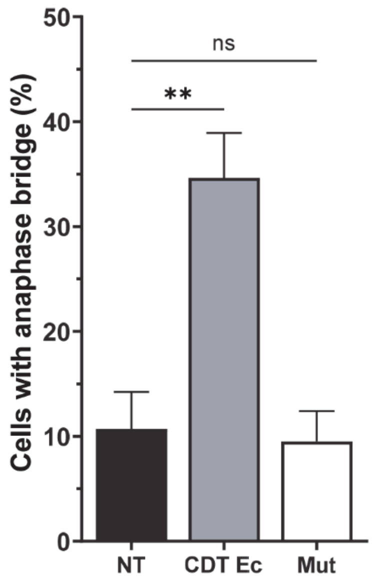

Supplementary Figure 1. CDT induces anaphase bridges. Quantification of anaphases with DNA bridge in HeLa cells treated or not (NT) with 100 pg/ml of wild type (CDT) or catalytically inactive CDT mutant (Mut) from *E.coli* for 24 hours. DNA was stained with DAPI. n> 45 anaphases were analyzed with a wide-field fluorescent microscope. (Mean ± SEM of three independent experiments). (**P < 0.01, *versus* non-treated (NT), one-way ANOVA followed by Dunnett's multiple comparison test was employed).

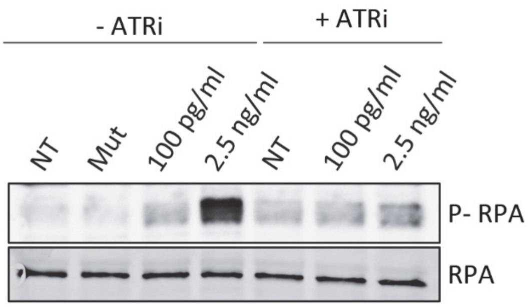

Supplementary Figure 2. CDT induces ATR activation. HeLa cells were treated or not (NT) with 100 pg/ml or 2.5 ng/ml of wild type or the catalytic inactive CDT mutant (Mut, 2.5 ng/ml) from *E.coli* with or without ATR inhibitor (ATRi, 5µM) for 24 hours. ATR activation was monitored by analyzing RPA32 phosphorylation on serine 33 by western blot. RPA32 is shown as a loading control.

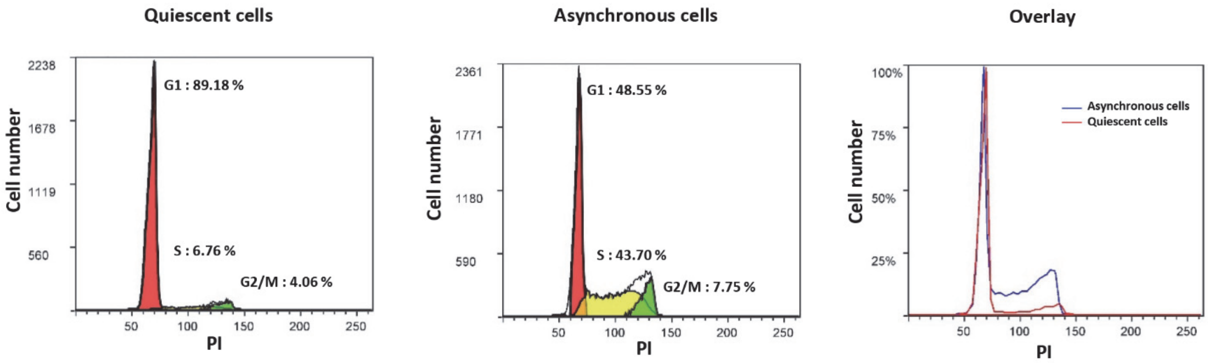

Supplementary Figure 3. Induction of quiescence in RKO cell line. Cell cycle analysis was performed in RKO cell line in proliferation or after induction of quiescence by confluence and serum starvation. DNA was stained with propidium iodide and the samples were processed using flow cytometry (MACSQuant, Miltenyi Biotec). At least 10,000 events were analyzed per sample using FlowLogic software. Watson logarithm was used to quantify the percentage of cell in each cell cycle phase (G1, S

and G2/M). On the right, an overlay of the histograms was generated to compare the cell cycle distribution in both conditions.
